# 2022 World of shipping Portugal: an international research conference on maritime affairs

**DOI:** 10.1186/s41072-023-00143-y

**Published:** 2023-05-06

**Authors:** Ana Cristina Paixão Casaca, Maria Amélia Ramos Loja

**Affiliations:** 1 ‘World of Shipping Portugal’, Parede, Portugal; 2 CIMOSM, ISEL - Centro de Investigação em Modelação e Optimização de Sistemas Multifuncionais, 1959-007 Lisbon, Portugal; 3 CIMOSM, ISEL - Centro de Investigação em Modelação eOptimização de Sistemas Multifuncionais, Instituto Politécnico de Lisboa, 1959-007 Lisbon, Portugal; 4 IDMEC, Instituto Superior Técnico – Universidade de Lisbon, Av. Rovisco Pais, 1, 1049-01 Lisbon, Portugal

**Keywords:** Maritime transport, Cold ironing, Container shipping, Maritime security, Digitalisation, Port community systems, Port governance, Maritime accidents

## Abstract

The COVID-19 pandemic has changed the world. It showed the possibility of running economic activities remotely, allowing people to learn how to have a more balanced life. As a result, electronic commerce flourished, and it is here to stay. However, only time will tell whether people will shop more online or return to traditional brick-and-mortar shopping. With more people at home, prevented from going out to a cinema, or a theatre, the service industry was severely impacted until governments relieved the confinement measures so that their economies returned to pre-pandemic levels. Certainly that container shipping was the winner of all shipping market segments, given the increased number of finished goods to be carried. When all seemed to return to normalcy, the Russian–Ukraine crisis complicated the economic and political environment. Maritime transport was affected in the Black Sea, and only after many weeks of negotiation did the parties involved reach the Ukraine grain deal enabling Ukraine to transport millions of tonnes of food through the Black Sea despite the ongoing conflict. The number of sanctions against Russia increased, and many countries and regions were forced to look for alternative sources of oil, oil by-products and gas, this time benefiting the gas and tanker shipping markets with increasing freight rates. Apart from this, the maritime industry is still facing extraordinary challenges. Endeavours are being made to accelerate industry decarbonisation, digitalisation and operations optimisation. The quest for finding alternative fuels to power the world fleet is there. For now, LNG and methanol are the most promising alternatives, with the possible installation of carbon storage units to mitigate carbon dioxide emissions into the atmosphere. These events draw the industry to deal with the market, technology, and regulatory challenges and risks whose outcome is yet to be seen. With this background, seven papers presented at the 2022 World of Shipping Portugal Conference, An International Research Conference on Maritime Affairs, 27–28 January, that took place online via CiscoWebex due to the Covid-19 pandemic were selected to be published in this Special Issue. They open the scope for new research areas and address essential aspects that contribute to the efficiency of the overall maritime sector.

Around the 2022 World of Shipping Portugal Conference dates, as the world population got vaccinated against COVID-19 and governments relieved the confinement measures so that their economies returned to pre-pandemic levels, many hurdles still prevented the world economy from returning to such conditions. Barriers still existed to those populations who lacked the complete vaccination programme, not to mention the reinforcement doses that some of us already had. The tourism sector was still in its revival infancy, although the number of people flying had increased. Two months after the Conference had taken place, when going to London, the aeroplane was fully booked, a good sign for aviation as airports were still depleted of people. We expected this positive trend to continue since numerous countries depend on tourism to achieve a positive balance of payments. Aviation was not the only sector that benefited from the increasing number of passengers flying. Numerous ancillary service activities, such as bars, restaurants, or agencies offering local sightseeing tours, also benefit. Moreover, tourism supports the goods markets and the manufacturing sectors since it promotes a more relaxing atmosphere in which we are more prone to shopping than in other moments of our lives. Fortunately, the situation has normalised, and at least in Lisbon, the city is in full swing.

Amidst all this, at the time of the Conference, the goods sector also faced unprecedented challenging moments. A complete revolution took place along manufacturing companies and other suppliers’ distribution channels, the extent of which is yet to be seen. Online shopping is here to stay. However, only time will tell whether people will shop more online or return to traditional brick-and-mortar shopping. According to UNCTAD, the statistics tell us that global trade reached US$ 24 trillion in 2022 and US$ 22 trillion in 2021, supported by subsidised pandemic restrictions and economic stimulus packages against US$ 17 trillion in 2020. Very high records if we consider the numerous and severe disruptions that supply chains have witnessed during 2021 and 2022. According to BIMCO, cargo volumes between Asia and North America increased by 27% in the first seven months of 2021 compared with pre-pandemic levels.

Factors leading to these disruptions derived from unpredictable demand swings had put pressure on supply chains, resulting in unprecedented shipping costs. In addition, the whole industry, including shipping companies, ports, shippers, and inland carriers, struggled with severe and widespread port congestion levels witnessed in many countries worldwide due to the COVID-19 pandemic. For example, on 9 January 2022, 109 ships waited to be berthed at Los Angeles and Long Beach, with berthing delays varying throughout the year and some ships having to wait up to 33 days to berth. All this resulted from a lack of (1) port workers, truck drivers, and port capacity, which resulted in high port inefficiencies, and (2) hinterlands infra- and superstructure, not to mention the container shortages, transport equipment like chassis and space on container ships. Fortunately, the witnessed congestion levels are a thing of the past; still, the contract negotiations between the Pacific Maritime Association and the International Longshore and Warehouse Union covering more than 22,000 dockworkers at 29 West Coast ports are still pending.

However, the most outstanding disruption witnessed was the temporary blockage of the Suez Canal in 2021 due to the Ever Given containership grounding, resulting in a halt of part of the maritime traffic using that maritime chokepoint. While highlighting the role of maritime transport in world trade and showing how the different raw materials, manufacturers and consumer markets are entwined, the Ever Given grounding also highlighted how fragile the existing supply chains were and still are.

Many companies managing their supply chains and logistics operations on a just-in-time basis were challenged with the need to change their supply chain strategies, i.e. from just-in-time to just-in-case, and implement more flexible and resilient supply chains capable of accommodating these shocks. Moreover, industries under pressure, such as the automotive one, had to stop due to the lack of components, namely semiconductors, and the issue of nearshoring or even reshoring came to the highlight even though such relocations may take time and go against the market globalisation principles witnessed in the last decades. However, according to Kearney’s this is becoming a reality. In its latest 2023 CEO survey, 96% of CEOs evaluate the potential to reshore their operations, against 78% in 2022, with most having decided to reshore or effectively reshored their operations.

As 90% of the goods traded internationally are carried by sea, the impact of these disruptions on maritime transport has been high. However, not all markets benefited from them. The tanker market witnessed severely unbalanced supply and demand conditions resulting in weak freight rates. Arthur Richier considered the year 2021 as a ‘tepid year’ compared with 2020, which was reported to be one of the most volatile years; according to him, all vessel classes experienced tonne-miles below 2019 averages for the whole of 2021. Most of the non-eco, non-scrubber crude tonnage was forced to operate at sub-zero levels for large parts of the year while the eco tonnage and those equipped with the exhaust gas cleaning technology achieved higher results, thus recouping part of their fixed operating expenses. The dry bulk market witnessed freight rates hitting decade-highs, subject to (1) supply chain bottlenecks and severe weather events, which exacerbated port congestion and disrupted terminal operations, and (2) global containerised trades.

However, indeed, the biggest winner was the container shipping market which witnessed unprecedented freight rates rate increases and surcharges and extraordinary profit levels due to the factors previously mentioned and others, such as high container dwell times inside ports both on exports and imports and increased blank sailings resulting in reduced service levels and carrier reliability. For example, on 21 September 2021, Shifl.com reported that the freight rate for a 40/HC from China to New York totalled US$19,500. Managing containers along their operational life cycle between origin and destination and consequent repositioning at the origin to be loaded again became a headache for the numerous logistics operators despite the entrance of 7.2 million TEU in the market, representing 14% of the global container equipment fleet in 2021. Moreover, this market segment has been forced to readjust the management of its capacity along the different trade routes it served, leading to the redeployment of containerships from one trade to another to overcome the fragile service reliability along the main trade routes, for instance, Asia–West Coast of North America. Meanwhile, some operators were chartering out containerships at US$200,000 a day for short-term employment, US$155,000 per day for a six-month charter, and owners selling a 6-year-old, 6865 TEU vessel for US$125 million.

Against all this, the shipping industry suffered from an unprecedented global crew-change crisis. The restrictions imposed on global travel left thousands of seafarers stranded on board ships, where they could neither sign off from their ships for their breaks nor join their ships to work. Many crews extend their contracts by several months, some of them not seeing land in 18 months. Situations of fatigue, depression and mental illness came to the public discussion as seafarers reported signs of them.

Among all this, the maritime industry is on its pathway to decarbonisation. However, this roadmap is complicated due to some countries’ economic interests and because many factors contribute to its success. Countries must reach a consensus to secure a global policy framework that includes taxing carbon emissions to create a level playing field and fund the research and development the industry desperately needs to decarbonise fully. The European Union ‘Fit for 55’ package, which contains a set of legislative proposals so that its climate, energy, land use, transport and taxation policies reach the European Green Deal’s objective to reduce the net greenhouse gas emissions by at least 55% by 2030 below 1990 levels and achieve climate neutrality by 2050, while being a good solution may not be a practical legislative proposal given the international character of the business and its complexity.

Moreover, shipping decarbonisation still faces technological challenges that the big industrial players, such as engine manufacturers, are still addressing; the zero-carbon vessel technology is still in its infancy. The technological challenge of developing engines capable of running on only one type of alternative fuel is still there, bearing in mind that some of these fuels may reduce ships’ cargo-carrying capacity and, consequently, vessels’ earnings to accommodate the fuel tanks without compromising ships’ safety, including their stability. Over the following years, the industry will surely rely on improving engine energy efficiency, with most ships built with dual-fuel engines to ply in the deep-sea trades, although the tri-fuel engine is also expected to dominate the mid-term transition.

Finding an alternative source of energy that will replace the current fossil fuel is still daunting. Without one alternative zero-emission fuel option in place, the industry needs to consider several viable alternative fuels to make the right decisions regarding the renovation of the fleet when the time comes. While liquefied natural gas is a transition fuel due to the methane slip, methanol appears to be a viable alternative. However, methanol production on a large scale to supply the world fleet requires considerable investments and resources into fuel production, supply chains, and a new or retrofitted fleet. Moreover, methanol production costs are currently high, and production volumes are low. This explains why A.P. Moller-Maersk has been securing yearly carbon–neutral e-methanol supplies to operate its carbon–neutral fleet. The production cost of hydrogen and hydrogen-derived fuels is still high. Hydrogen does not exist naturally and is not easy to store or transport because of its low volumetric energy density and small molecular size. The production of hydrogen and hydrogen-derived fuels also needs considerable resources, which are available in some regions of the globe and require well-designed and implemented distribution chains between production and consumption markets. Therefore, lowering their costs is possible only if the demand increases to scale up production, particularly in green hydrogen. Ammonia, on the other hand, as an alternative shipping fuel, mainly if it concerns green ammonia, is seen as the potential long-term solution for running zero-carbon maritime value chains; however, its handling raises concern due to its toxicity level.

The alternative could be vessels’ retrofitting; however, this may not be an option given its high costs. As a short-term strategy, shipping companies must trade off the ships’ age against their earning capacity over the remaining years of their life to see if such an investment is feasible. However, finding shipyards to carry out such work may be difficult given the limited capacity and order book size. As of 28 February 2022, Mediterranean Shipping Company, Evergreen Line, CMA CGM Group and Wan Hai Lines accounted for 232 containerships. Almost 14 months later, BIMCO claims that the previous ten quarters have witnessed 8.61 million TEU being contracted, thus resulting in significant fleet growth.

Besides, industry decarbonisation will benefit from other disciplines, such as digitalisation, smart operations, voyage optimisation, and machine learning, that contribute to better industry performance, albeit at higher freight rates, due to the price of alternative fuels. Creating green corridors also helps decarbonise the shipping industry if shippers are prepared to pay the high freight rates incurred with cargo moving along zero-emission trade routes. Vessels fitted with engines running on green methanol are expected to be 10–15% more expensive since the alternative fuel costs more than twice the price of conventional bunker fuel. Overall, the decarbonisation of the shipping industry is more than a policy or technological issue. It also concerns a change of mentality. While keeping the knowledge learned over the years, the zero-carbon transition requires shipowners to adopt new business models and be disruptive to the past. Shipping companies’ future growth strategies must consider environmental, social, and governance standards as banks’ and financial institutions’ criteria for screening investments become stricter. With capital loans becoming more expensive than before, the industry has an opportunity to remove the existing substandard fleet since shippers will also be accountable for the pollution they cause.

The current Russia–Ukraine crisis, which results from the one that began in 2014 with the annexation of Crimea by the Russian Federation, impacted the global economy with inflation and rising consumer prices. The first impacts were witnessed in the energy markets, with energy prices soaring almost a week after Russia invaded Ukraine. The oil price peaked at nearly US$114 per barrel, the Dutch April gas contract hit a new record high of €185 per megawatt-hour, trading 41% higher, and the European coal prices for 2023 rose to a record $260.5 a tonne. However, the economies of both countries will suffer even more from the negative impacts of this crisis. While Russia is suffering from strict international sanctions affecting its international and domestic economies, for instance, by removing its banks from the SWIFT system or blocking about 429 internationally trading vessels over 10,000 dwt flagged, owned, or operated by Russian interests from entering the European Union, British and Canadian ports, the Ukraine economy is destroyed because of the massive attacks caused by the Russian army.

The Russian military action in February 2022 initially reduced maritime trade in the Black Sea by blocking Ukraine’s ports, trapping some 20 million tonnes of grain. In addition, Ukraine is a global sunflower, maise, wheat, and barley exporter. This forced countries having to find alternative sources of Ukrainian ores, Ukrainian and Russian grains (wheat and corn) and Russian crude oil. However, this situation appears to have been, at least, temporarily resolved with the Ukraine grain deal that has enabled Ukraine to transport millions of tonnes of food through the Black Sea despite the ongoing conflict. Ultimately, these events will contribute positively to the global earnings of some shipping industry sectors, namely the tanker and gas markets. Replacing supply sources means longer distances travelled by goods, whatever their nature, resulting in increasing ton-mile, more ship capacity and higher freight rates. History has shown that wars and similar conflicts have benefited the industry, even temporarily. According to Glenn Koepke, ocean rates could double or triple due to the invasion, from $10,000 per 40-foot container to $30,000. Moreover, the 200 ships waiting to cross the Kerch Strait, connecting the Black and the Azov Seas, can increase these rates.

Within this context, seven papers were chosen to be part of this Special issue.

*Pruyn and Willeijns* deal with cold ironing. The authors claim cold ironing can reduce ship exhausts’ impact in densely populated areas. Therefore, the paper identifies existing systems and solutions and analyses their applicability in the tanker fleet and terminals, which in the case of tankers, it is almost non-existent. An integrated economic model consisting of two sub-models was used to carry out the analysis. One sub-model addressed terminal decisions to a cold ironing price; the second sub-model established the vessel costs and savings.

*Martius *et al*.* address repositioning empty containers from surplus to deficit regions. However, the authors claim that the container availability prediction models are limited to individual regions and are characterised by high temporal aggregation. In order to address these limitations, the paper proposes two novel approaches based on machine learning and probabilistic techniques to predict the future weekly availability of empty containers for more than 280 locations worldwide.

*Sackey *et al*.* analyse maritime insecurity’s impact on seafarers’ and marine professionals’ lives, the environment and property. Furthermore, it investigates the economic cost of traditional maritime crimes within the scope of the African Continental Free Trade Area. To achieve this, the case study approach, in which the Gulf of Guinea region was observed for 3 years, was supported by various remote interviews and online surveys. The study indicates the need for a community-based approach to surveillance.

*Mthembu and Chasomeris* analyse port community systems in South Africa. The authors claim that port community systems productivity, efficiencies, and competitiveness whilst improving the port’s attractiveness by connecting port users and supply chain participants. The paper uses the soft systems methodology supported by interviews and workshops. It presents a framework for implementing port community systems.

*Plomaritou and Jeropoulos* examine the role of digitalisation’s the chartering business to enhance efficiency in the shipping business and the role of the e-bill of lading in the bulk and liner markets. The research adopts a qualitative case study approach. The paper shows that while digital technologies are advantageous in the chartering business, many legal barriers still need to be overcome.

*Andersen *et al*.* bring together port governance and stakeholder theory to understand stakeholder relations changes to constructing a new container terminal developed and operated by Meridian Port Services in the Port of Tema, Ghana. Using focus groups, descriptive statistics, and a series of qualitative and open-ended interviews, the paper addresses hybrid port governance systems. Moreover, it provides insights into the different strategic choices’ implications and how changes progress.

Finally, *Dominguez-Péry *et al*.* investigated risk indicators in maritime accidents and how they are addressed when reported. In order to achieve this, the paper considers International Maritime Organisation accident reports from 2011 to 2000, which indicated that human error continues to be the top-cited cause of accidents. Using a data-driven approach, statistical and advanced text-mining techniques, the paper proposes the Accident Maritime Ecosystem framework, which incorporates individuals, the ship organisation, the internal ship ecosystem, the external ship ecosystem and the global maritime ecosystem.

We thank the Journal of Shipping and Trade for accepting this Special Issue and China Merchants Energy Shipping for waiving this Special Issue process charges.

Greetings from Lisbon.

Ana Cristina Paixão Casaca and Maria Amélia Ramos Loja.

## Data Availability

Not applicable.

